# Draft Genome Sequence of *Pedobacter* sp. Strain Hv1, an Isolate from Medicinal Leech Mucosal Castings

**DOI:** 10.1128/genomeA.01469-15

**Published:** 2015-12-17

**Authors:** Brittany M. Ott, Lidia Beka, Joerg Graf, Rita V. M. Rio

**Affiliations:** a Department of Biology, West Virginia University, Morgantown, West Virginia, USA; b Department of Molecular and Cell Biology, University of Connecticut, Storrs, Connecticut, USA; c Institute for Systems Genomics, University of Connecticut, Storrs, Connecticut, USA

## Abstract

The *Pedobacter* sp. Hv1 strain was isolated from the medicinal leech, *Hirudo verbana,* mucosal castings. These mucosal sheds have been demonstrated to play a role in horizontal symbiont transmission. Here, we report the draft 4.9 Mbp genome sequence of *Pedobacter* sp. strain Hv1.

## GENOME ANNOUNCEMENT

The genus *Pedobacter* was reclassified from the genus *Sphingobacterium* in 1998 (1) due to various phenotypic variations. Members of the *Pedobacter* genus have been described from water and soil samples, as well as from the microbiotas of nematodes, dung beetles, and mosquitoes (2–4). Recently, strain Hv1 was identified as the most abundant bacterium within the mucosal casts of medicinal leeches (*Hirudo verbana*) (5). Here, we present a draft genome of *Pedobacter* sp. Hv1 strain.

The genome of *Pedobacter* Hv1 was sequenced using the Illumina MiSeq (1,880,901 paired-end reads). Raw reads were assembled using the CLC Genomics Workbench (Aahrus, Denmark), yielding 30 total contigs with lengths of >0.5 kb and an *N*_50_ of 665,494 bp. Due to low coverage, three contigs were immediately eliminated. The *oriC* was detected using Ori-Finder (6, 7) in Contig 12 (positions 65,484 to 65,930). An additional assembly was generated using AbySS (8) with varying k-mer lengths, which were aligned with the CLC assembly using Mauve (9, 10). However, the gaps could not be resolved. BLASTn detected 4 contigs with ~100% sequence identity and 0 gaps compared to regions in larger contigs. Subsequently, Tandem Repeats Finder (11) did not identify long terminal repeats typically associated with mobile elements, and the lack of obvious G+C skew eliminates recent gene duplication events. Mapping reads with BWA (Burrow-Wheeler Alignment tool) (12) and IGV (Integrative Genomics Viewer) (13, 14) eliminated these contigs due to high probability of an assembly error, resulting in 23 total contigs with lengths >0.85 kb. The updated contigs totaled 4,900,269 bp with an average coverage of 78×; *N*_50_ of 666,494 bp. The G+C content is 37.2%.

Functional annotation of the assembled genome was performed with RAST 2.0 (15, 16), and manual annotation and curation, which predicted 4,421 protein encoding genes and 38 RNAs. RNAs were further classified using tRNAscan-SE-1.23 (17), which predicted 35 tRNA genes (including tRNAs for all 20 amino acids, with 4 each for leucine and arginine), and the RNAmmer 1.2 server (18), which identified one 5S, one 23S, and one 16S rRNA gene. Genes related to the utilization and biosynthesis of carbohydrates, including N-acetylglucosamine, mannose, and trehalose decomposition, suggests the capability of adapting to varying metabolites. Hv1 contains a number of loci involved in osmotic and oxidative stress response, such as superoxide dismutase [Cu-Zn] precursor, cytochrome c551 peroxidase, and the universal stress protein family 4.

The Hv1 genome contains loci involved in type I, III, and IV secretions systems. Of particular interest, given the diverse microbiota from which Hv1 was originally isolated, is the presence of the conjugative transposon (e.g., various *tra* loci), associated with the type IV secretion system and the transfer of DNA from environmental sources (19). Interestingly, this operon is missing in the closest, fully sequenced relative, *Pedobacter heparinus* (20) (RAST, closest neighbor tool; similarity score, 542), while the more distantly related *P. saltans* (21) houses the same genetic components of the *tra* operon (with the exception of *tra*G and *tra*P).

### Nucleotide sequence accession numbers.

This whole-genome shotgun project has been deposited at DDBJ/EMBL/GenBank under the accession no. LLWP00000000. The version described in this paper is version LLWP01000000.
